# The Emerging Neuroscience of Intrinsic Motivation: A New Frontier in Self-Determination Research

**DOI:** 10.3389/fnhum.2017.00145

**Published:** 2017-03-24

**Authors:** Stefano I. Di Domenico, Richard M. Ryan

**Affiliations:** ^1^Institute for Positive Psychology and Education, Australian Catholic UniversityStrathfield, NSW, Australia; ^2^Department of Clinical and Social Sciences in Psychology, University of RochesterRochester, NY, USA

**Keywords:** curiosity, dopamine, flow, intrinsic motivation, PLAY system, salience network, SEEKING system, self-determination theory

## Abstract

Intrinsic motivation refers to people’s spontaneous tendencies to be curious and interested, to seek out challenges and to exercise and develop their skills and knowledge, even in the absence of operationally separable rewards. Over the past four decades, experimental and field research guided by self-determination theory (SDT; Ryan and Deci, 2017) has found intrinsic motivation to predict enhanced learning, performance, creativity, optimal development and psychological wellness. Only recently, however, have studies begun to examine the neurobiological substrates of intrinsic motivation. In the present article, we trace the history of intrinsic motivation research, compare and contrast intrinsic motivation to closely related topics (flow, curiosity, trait plasticity), link intrinsic motivation to key findings in the comparative affective neurosciences, and review burgeoning neuroscience research on intrinsic motivation. We review converging evidence suggesting that intrinsically motivated exploratory and mastery behaviors are phylogenetically ancient tendencies that are subserved by dopaminergic systems. Studies also suggest that intrinsic motivation is associated with patterns of activity across large-scale neural networks, namely, those that support salience detection, attentional control and self-referential cognition. We suggest novel research directions and offer recommendations for the application of neuroscience methods in the study of intrinsic motivation.

*Intrinsic motivation* refers to the spontaneous tendency “to seek out novelty and challenges, to extend and exercise one’s capacity, to explore, and to learn” (Ryan and Deci, 2000, p.70). When intrinsically motivated, people engage in an activity because they find it interesting and inherently satisfying. By contrast, when extrinsically motivated, people engage in an activity to obtain some instrumentally separable consequence, such as the attainment of a reward, the avoidance of a punishment, or the achievement of some valued outcome. Early evidence for the distinction between these types of motivation came from experimental studies demonstrating that tangible rewards can undermine intrinsic motivation (Deci, 1971). That is, contrary to the ideas that intrinsic and extrinsic motivation are additive or synergistically positive (e.g., Atkinson, 1964; Porter and Lawler, 1968), studies show that people experience less interest and exhibit less spontaneous engagement with activities for which they were initially intrinsically motivated after receiving tangible rewards for performing the activities (Deci et al., 1999).

*Self-determination theory* (SDT; Ryan and Deci, 2000, 2017) has emerged as the principle framework for the study of intrinsic motivation. Intrinsic motivation is frequently assessed behaviorally in terms of freely pursued activities, and experientially through self-report questionnaires that probe the reasons for one’s engagement with activities, as well as specific affective states such as interest, curiosity and fun. Intrinsic motivation has also been assessed in the laboratory through the coding of specific exploratory and manipulatory behaviors and facial displays of interested engagement (Reeve and Nix, 1997). Since the earliest demonstrations of the undermining effect, many experimental and field studies have found intrinsic motivation to be associated with enhanced learning, performance, creativity, and affective experience. Further, a large body of research within SDT has examined the situational factors (e.g., types of rewards, feedback, communication styles) that undermine or facilitate the expression of intrinsic motivation (Ryan and Deci, 2017). These studies have made it clear that although intrinsic motivation is a lifelong psychological growth function, by no means is its expression automatic; rather, intrinsic motivation depends on ambient supports for basic psychological needs, especially those for competence (feeling effective) and autonomy (feeling volitional).

Despite being a longstanding topic within the field of motivation, only recently have researchers begun to use neuroscience methods to examine intrinsic motivational processes (Ryan and Di Domenico, 2016). The use of neuroscience methods is an important new frontier for intrinsic motivation research for at least three interrelated reasons. First, to state the obvious, experience and behavior are mediated by the brain and a complete account of intrinsic motivation therefore requires an understanding of the neural systems that support it. Second, neuroscience affords the examination of internal processes that are not accessible by self-reports of experience or behavioral observations. A neuroscience of intrinsic motivation therefore promises new insights that introspective and behavioral methods alone cannot afford. Finally, neuroscience methods can be used to investigate motivational processes at a higher level of resolution than experiential and behavioral methods. Neuroscience methods therefore have the potential to refine conceptual accounts of intrinsic motivation by articulating the granular processes that comprise it. In a relevant discussion, Ochsner (2007; p.51) stated that, “The combination of multiple streams of data allows researchers to converge on theoretical explanations that are robust and flexible and are not tied to a single specific experimental methodology”. Intrinsic motivation would seem to be an especially ripe topic for neuroscience precisely because of the large body of empirical data that has already been garnered at the experiential and behavioral levels of analysis.

Our purpose of this review article is to survey the progress of neuroscience research on intrinsic motivation. Because intrinsic motivation is not a uniquely human capacity (Harlow, 1953; Wilson, 2000; Ryan and Deci, 2017) we review conceptual developments in the comparative affective neurosciences (Panksepp, 1998; Panksepp and Biven, 2012) that inform the concept of intrinsic motivation. Such considerations are essential for appreciating intrinsic motivation as a basic organismic capacity and for helping to clarify its unique components in humans (Ryan and Di Domenico, 2016). Building upon these insights, we map the phenomenology of intrinsic motivation onto the neural substrates of motivational processes that are encompassed by intrinsic motivation. Against the backdrop of these preliminary ideas, we then review recent studies that have examined the neural correlates of intrinsic motivation. To anticipate our main conclusions, affective neuroscience suggests that human intrinsic motivation is based in ancient mammalian systems that govern exploration and play. Neuroimaging studies, which have up to now focused on curiosity and mastery tendencies, indicate that intrinsically motivated states are subserved by neural regions that are central to dopamine systems. These studies also hint at the possible role of dynamic switching between large-scale brain networks involved in salience detection, attentional control and self-referential cognition. On the basis of these ideas, we suggest novel research directions and offer recommendations for the application of neuroscience methods in the study of intrinsic motivation.

## Intrinsic Motivation: An Organismic Growth Process

Long before Deci’s (1971) experiments concerning intrinsic motivation in humans and its undermining by rewards, Harlow (1950) documented this effect in rhesus monkeys. He coined the term *intrinsic motivation* to describe his observation that these primates would persist in playing with mechanical puzzles even in the absence of external rewards. Indeed, he observed that the introduction of rewards for playing led these primates to decrease their spontaneous manipulative explorations, relative to those not exposed to external rewards. These and related observations of spontaneous exploratory and play behaviors defied some behaviorist views that intentional behaviors are invariably controlled by reinforcement contingencies within the environment (e.g., Skinner, 1953).

Early work with both primates and rats also exposed some limitations of empirical drive theory (Hull, 1943), which asserted that motivated behaviors aim to reduce internal drives that stem from physiological need deficits. Because intrinsic motivation often ensues in the absence and, on occasion, independent of such deprivations, it was poorly explained by traditional drive reduction accounts (White, 1959). Early attempts to amend drive theory led researchers to postulate the existence of various exploratory drives as the basis for seemingly spontaneous curiosity, exploratory and manipulatory behaviors (e.g., Butler, 1953; Harlow, 1953; Montgomery, 1954; Myers and Miller, 1954). Apart from its lack of parsimony, this “drive-naming” approach could not be reconciled with the observations that exploratory activities do not resemble consummatory responses and that animals often behave to *increase* rather than *decrease* such exploratory drives (White, 1959; Deci and Ryan, 1985). As we shall see, these points are respectively echoed in the contemporary research on the role of dopamine in motivation, particularly by Berridge (2007) distinction between incentive “wanting” and consummatory “liking” and by Panksepp’s (1998) work on the mammalian SEEKING system.

Variants of psychodynamic drive theory (Freud, 1927/1960) proved similarly inadequate. For example, Fenichel (1945) proposed that exploratory and mastery behaviors are driven by the desire to reduce anxiety in the face of novel stimuli. A revision of this hypothesis may be approximated from the perspective of Gray and McNaughton’s (2000) septo-hippocampal theory of anxiety. An extensive program of research has established that novel stimuli—on an *a priori* basis—represent potential sources of both punishment and reward, elicit tendencies for both avoidance and approach, and therefore often arouse anxious uncertainty and prompt cautious investigatory behaviors. The investigatory behaviors instigated by the septo-hippocampal system include risk assessment and scanning the environment and one’s memory to resolve the motivational conflict, that is, to compute whether approach or avoidance should predominate.

Intrinsically motivated curiosity, exploration and mastery behaviors, however, pertain to specific types of novel stimuli, namely, those that present optimal challenges or optimal inconsistencies with one’s extant knowledge and that accordingly energize tendencies to approach (White, 1959; Csikszentmihalyi, 1990; Loewenstein, 1994; Ryan and Deci, 2017). Consistent with the work of Gray and McNaughton (2000) intrinsic motivation researchers have long noted that whereas too much novelty relative to a person’s skill and knowledge produces anxiety, too little novelty produces to boredom. During intrinsic motivation, feelings of interest and positive excitement predominate over both anxiety and boredom. Indeed, such exploratory states entail searching for novelties and challenges and, moreover, acting on the world to elicit novelties and to discover new problems (Harlow, 1953; White, 1959; Deci and Ryan, 1985). These observations indicate that intrinsically motivated exploratory and mastery behaviors are primarily energized by interest and appetitive mastery tendencies, not anxiety reduction.

Given the shortcomings of operant behaviorism and drive theory in regards to intrinsic motivation, White (1959) proposed *effectance motivation* as a general behavioral and developmental propensity of many organisms. Seemingly prescient of later developments in the affective neurosciences (e.g., Panksepp, 1998; Panksepp and Biven, 2012), White (1959) argued that effectance motivation is inherent to the activity of the central nervous system and described it as “what the neuromuscular system wants to do when it is otherwise unoccupied (e.g., by strong homeostatic drives) or is gently stimulated by the environment” (p.321). According to White (1959), the satisfactions associated with the effectance motive are not tied to consummatory activities, but are instead intrinsic to the arousal and maintenance of the activities that stem from it. Along similar lines, DeCharms (1968) proposed that intrinsic motivation is based in people’s “primary propensity” to experience themselves as causal agents, that is, to experience their own actions as having an *internal perceived locus of causality*. DeCharms’s (1968) insightful theorizing helped set the stage for the earliest experiments on the undermining effect as it suggested that external enticements and pressures that detract one from experiencing oneself as the center of initiation of their own behaviors—that undermine autonomy—can diminish intrinsic motivation (Deci and Ryan, 1985).

By the mid-1980s, numerous studies had examined the effects of various situational factors on the expression of intrinsic motivation (Deci and Ryan, 1985). This research indicated that events like the provision of positive feedback (e.g., Fisher, 1978; Boggiano and Ruble, 1979; Ryan, 1982) and choice (e.g., Zuckerman et al., 1978) enhanced intrinsic motivation and that negative feedback (e.g., Deci and Cascio, 1972; Vallerand and Reid, 1984), deadlines (e.g., Amabile et al., 1976), and other external impositions (e.g., surveillance; Lepper and Greene, 1975) generally diminished intrinsic motivation. To account for the diversity of findings from these and other studies, Deci and Ryan (1985), drawing on the ideas of White (1959) and DeCharms (1968), proposed that intrinsic motivation is a lifelong psychological growth function that is based in the basic psychological needs for competence and autonomy. *Competence* refers to feelings of effectance, the sense of growing mastery in activities that are optimally challenging and that further develop one’s capacities. *Autonomy* refers to an experience of volition and integrity, the sense that one’s behavior is authentic and self-organized rather than internally conflicted and pressured or externally coerced. Within SDT, competence and autonomy are seen as essential elements in people’s active propensities to seek out challenges, to be curious and interested, and to develop and express their capacities: when these needs are supported, intrinsic motivation may ensue; when these needs are thwarted, intrinsic motivation is undermined (Ryan and Deci, 2017).

In terms of both evolution and development, intrinsic motivation confers many adaptive consequences for organisms (Ryan and Deci, 2017). For example, intrinsic motivation exposes organisms to novel situations and therefore occasions the development of diverse skills and competencies to cope with uncertain future situations. Intrinsic motivations are particularly important for those species that have a protracted period of postnatal development and occupy complex habitats (Wilson, 2000). In this vein, Deci and Ryan (2000, p.252) pointed out that:

If people did not experience satisfaction from learning for its own sake (but instead needed to be prompted by external reinforcements) they would be less likely to engage the domain-specific skills and capacities they inherited, to develop new potentialities for adaptive employment, or both … for instance, by aiding in the discovery of alternative food sources, mapping the complexities of game migrations, or taking interest in skills, rituals, and social rules transmitted by other group members.

Extending this evolutionary thinking, Ryan and Hawley (2016) reviewed empirical evidence that competence and autonomy satisfactions supply proximal supports for intrinsically motivated activities even when the adaptive consequences of such activities are not the phenomenal aims of the individuals enacting them.

At the level of personality functioning, intrinsic motivation provides the impetus for individuals to learn about particular subject areas and to differentiate their interests, fostering the development of personal identities that confer a sense of authenticity, meaning, and purpose (Deci and Ryan, 1985; Ryan and Deci, 2012). For example, meta-analyses and field studies point to intrinsic motivation as perhaps the most important form of motivation in school achievement (e.g., Taylor et al., 2014; Froiland and Worrell, 2016). In a related vein, Peterson (1999) argued that the dedicated and courageous pursuit of one’s interests optimizes personality development by incrementally exposing one to new ideas and challenges, thereby preventing ideological rigidity and fostering learning, growth, and meaning in life. Indeed, various scholars have proposed that intrinsically motivated self-examination plays a key role in the development of the highest human virtues, including wisdom (e.g., Habermas, 1972; Csikszentmihalyi and Rathmunde, 1990; Vervaeke and Ferraro, 2013).

### Neuroethological Perspectives on Mammalian Exploration: A Starting Point for Conceptualizing Intrinsic Motivation in the Brain

We previously pointed out (Ryan and Di Domenico, 2016) that the concept of intrinsically motivated exploration is consistent with the “affective neuroethological” perspective of Panksepp and colleagues (Panksepp, 1998; Ikemoto and Panksepp, 1999; Alcaro et al., 2007; Alcaro and Panksepp, 2011; Panksepp and Biven, 2012). These researchers have argued that mammals are hardwired with a general-purpose *SEEKING system* that energizes many types of foraging and exploratory activities. Although the SEEKING system does service homeostatic imbalances and is responsible for energizing learned appetitive behaviors, it continuously operates to keep animals in a state of exploratory engagement with their environments. That is, the SEEKING system is believed to function as an objectless appetitive system—a “goad without a goal”—until the exploratory disposition it produces leads to the discovery and learning of useful regularities.

The SEEKING system is a spontaneous, unconditioned behavior generator that takes animals to places, actively and inquisitively, where associated learning mechanisms allow them to develop knowledge structures, to guide their foremost evolutionary action tools (inbuilt emotional systems) to create more structures—more higher mental processes—which facilitate survival (Panksepp and Biven, 2012, p.135).

It is worth pointing out that even within radical behaviorism this inherent activity of organisms could not be fully ignored, though it was marginalized by Skinner’s concept of “the operant”. Skinner acknowledged that organisms do “operate” on their environments, but regarded such exploratory activities as random behaviors that come under the control of external reinforcement.

The core structures that comprise the SEEKING system in the rat are the ventral tegmental area (VTA), the nucleus accumbens (NAcc), the ventromedial prefrontal cortex (VMPFC), and the dopaminergic projections originating from the VTA that innervate these areas (Panksepp, 1998; Panksepp and Biven, 2012). These regions are frequently called the “brain reward network” because, as Olds and Milner (1954) discovered, rats will learn to instrumentally obtain electrical stimulations in this area. However, the invigorated searching and sniffing following such electrical stimulations look like states of invigorated curious exploration rather than states of calm satiation (see White, 1959): “The most dramatic observation…is that animals getting this kind of brain stimulation frantically explore their environments, taking notice of all the new stimuli they encounter” (Panksepp and Biven, 2012, p.126). These basic SEEKING urges are elaborated into more complex forms of exploration in behaviorally and cognitively sophisticated animals: our dexterity affords the manipulation and exploration of complex objects and our cognitive faculties afford interest in ideas, abstract objects and possibilities that we can explore and manipulate with our minds. The SEEKING system is thus believed to energize “many mental complexities that humans experience as persistent feelings of interest, curiosity, sensation seeking and, in the presence of a sufficiently complex cortex, the search for higher meaning” (Panksepp, 1998, p.145).

The first experimental studies on intrinsic motivation were conducted on nonhuman animals (Harlow, 1950) and it is therefore fitting that the first insights on the neurobiology of intrinsic motivation have been derived in animal research. Although generalizations based on animal research must be made with caution, affective neuroscience suggests that human intrinsic motivation is an elaboration of ancient mammalian motivations for exploratory SEEKING. The affective neuroethological point of view from which this system is conceptualized dovetails the organismic perspective from which SDT developed (Ryan and Deci, 2017). It is remarkable and telling that independent lines of research stemming from such methodologically diverse traditions should converge on similar points of view.

## Related Perspectives on Intrinsic Motivation

Aspects of intrinsic motivation have also been examined from perspectives other than SDT. Because some of the empirical studies that we review in upcoming sections are based on these related topics, we briefly summarize these perspectives here to note similarities and differences with SDT. We also briefly review topics that bear important conceptual relations to intrinsic motivation and note the utility of these for helping to inform the emerging neuroscience of intrinsic motivation.

The close relation between SDT’s concept of intrinsic motivation and Csikszentmihalyi (1990) concept of *flow* has been noted for a long time (Deci and Ryan, 1985, 2000). Flow refers to experiential states of total absorption, optimal challenge, and non-self-conscious enjoyment of an activity. Like intrinsic motivation, when people experience flow, the satisfactions they experience are inherent to the activity itself and their behavior is “autotelic” (*auto* = self, *telos* = goal) or performed for its own sake. Like SDT, flow theory emphasizes the phenomenology of intrinsic motivation. Flow theory is particularly articulate in its description of the optimal challenges and ensuing competence satisfactions associated with intrinsic motivation. For example, Nakamura and Csikszentmihalyi (2014; p.90) describe the flow state as the subjective experience of engaging “just-manageable challenges by tackling a series of goals, continuously processing feedback about progress, and adjusting action based on this feedback”. However, apart from recognizing the autotelic (i.e., intrinsically motivating) aspects of flow activities, flow theory does not formally recognize autonomy as an essential component of flow (Deci and Ryan, 2000).

Loewenstein (1994) proposed an “information-gap” hypothesis of curiosity according to which curiosity arises when people experience a discrepancy between what they know and what they want to know. Although this knowledge discrepancy is supposedly experienced as aversive, satisfying curiosity is pleasurable and people therefore voluntarily seek to elicit curiosity. There are some obvious links between SDT and Loewenstein’s (1994) information-gap hypothesis of curiosity. First, feelings of curiosity are regularly referenced in descriptions of intrinsic motivation within SDT and Loewenstein (1994; p.87) correspondingly described curiosity as “an intrinsically motivated desire for specific information”. Second, both intrinsic motivation and curiosity seeking are processes that describe types of self-directed learning. Finally, although Lowenstein’s theory does not formally include the concept of autonomy, his notion of what constitutes an “information-gap” is well-aligned with SDT’s notion of competence. Specifically, one way to conceptualize information-gaps in knowledge is in terms of optimal incongruities between one’s extant knowledge structures and the unknown (Deci and Ryan, 1985). Intrinsically motivated activities, activities that are energized by the need for competence and that entail orienting toward novel stimuli and optimal challenges, can thus be seen as a process of continually seeking and reducing information-gaps in knowledge.

Perhaps the most notable divergence between SDT and Loewenstein’s account concerns his description of curiosity as a consummatory, drive-reduction process—i.e., the closure of information gaps. A close variant of this discrepancy between organismic and drive-theory accounts of intrinsic motivation was resolved in the earliest critiques of the drive-naming approach to intrinsically motivated exploration. Both White (1959) and Deci and Ryan (1985) pointed out that while curiosity for particular objects or places may satiate the tendency to explore those particular objects or areas, the tendency to explore itself is not satiated. Thus, SDT’s organismic account of intrinsic motivation and Loewenstein’s (1994) drive-reduction account of curiosity seeking can be reconciled by recognizing that curiosity is a more delimited phenomenon subsumed by intrinsically motivated exploration. Piaget (1971), in his organismic account of cognitive development, expressed a similar view. He proposed that cognitive-behavioral schemata possess *inherent functions* to assimilate new information and to elaborate pre-existing skills, inherent functions that can be productively described as being intrinsically motivated (Ryan and Deci, 2017). Piaget (1971) thus saw curiosity as a continual process that “goes through various steps, in the sense that whenever one problem is solved, new problems are opened up. These are new avenues for curiosity” (Evans, 1973, pp.68–69).

A concept related to intrinsic motivation has also emerged within the “Five-Factor” or “Big Five” model of personality research (John et al., 2008; McCrae and Costa, 2008). Specifically, DeYoung (2010, 2013) has argued that the higher-order trait *plasticity* (i.e., the shared variance of extraversion and openness/intellect) represents stable interindividual differences in people’s exploratory tendencies. Apart from the obvious difference that intrinsic motivation refers to a *motivational* *state*, whereas plasticity refers to *dispositional trait*, these two phenomena have some notable features in common. Like intrinsic motivation, plasticity entails being “actively engaged with the possibilities of the environment, both generating and attending to novel aspects of experience” (DeYoung, 2010, p.27, and although plastic exploration has not been formally described using the concept of autonomy, people high in plasticity are hypothesized to “desire exploration for its own sake (i.e., they treat it as a goal in itself) and engage in it even at times when exploration will not obviously further their goals” (DeYoung, 2013, p.8). These conceptual links between plasticity and intrinsic motivation are important because recent years have seen a marked increase in the field’s understanding of the neurobiology of plasticity, most specifically, its association with dopamine (DeYoung, 2013). These insights inform some of the ideas in the current presentation.

## Mapping Phenomenology to Brain Function: Toward a Neurobiological Model of Human of Intrinsic Motivation

The biggest challenge facing researchers who wish to examine the neural substrates of intrinsic motivation is the absence of an overarching neurobiological framework with which to derive and test specific hypotheses. Exploratory studies, though potentially useful for advancing research in novel directions when conducted with suitably large samples, typically afford lower statistical power and are therefore prone to both Type I errors (false positives) and Type II errors (false negatives). This limitation of exploratory research is especially problematic in neuroimaging studies that do not specify *a priori* regions of interest and need to correct for multiple statistical tests when comparing neural activity across multiple brain regions (Allen and DeYoung, 2016). In the absence of a guiding theory, it is also difficult to design experimental paradigms that are optimally suited to examine specific components of intrinsic motivation.

Recognizing that even a preliminary neurobiological account of intrinsic motivation could facilitate theory-driven research and provide a useful vantage point for aligning the disparate empirical studies to date, we offer an initial iteration by mapping the phenomenology of intrinsic motivation to the neural substrates of motivational processes that are encompassed by intrinsic motivation. We organize these ideas in the form of summary propositions. Against the backdrop of these propositions, we review studies that have examined the neural correlates of intrinsic motivation.

### Proposition I: Intrinsic Motivation is Supported by Dopaminergic Systems

Intrinsic motivation is a complex cognitive, affective, and behavioral phenomenon that is likely mediated by multiple neural structures and processes. For this reason, a useful point of entry for elucidating the neurobiology of intrinsic motivation is to consider the broad neurotransmitter systems that may underlie it.

Three lines of evidence suggest that dopamine is a key substrate of intrinsic motivation. First, as the review above suggests, intrinsic motivation in humans is an elaboration of the exploratory activities subserved by the mammalian SEEKING system, and dopamine is central to the neurochemistry of this system (Panksepp, 1998; Panksepp and Biven, 2012). Second, like intrinsic motivation, dopamine is associated with increased positive affect, cognitive flexibility, creativity (Ashby et al., 1999), behavioral persistence (Salamone and Correa, 2016), and exploration in the face of novelty (DeYoung, 2013). Importantly, the positively affective states associated with dopamine reflect energized appetitive “wanting” not consummatory “liking”, the hedonic effects of which are mediated by opioids (Berridge, 2007; Kringelbach and Berridge, 2016). Third, there is some evidence of a direct link between intrinsic motivation and dopamine. Using positron emission tomography, de Manzano et al. (2013) found that people who are disposed to experience intrinsically motivated flow states in their daily activities have greater dopamine D2-receptor availability in striatal regions, particularly the putamen. This finding suggests that people’s capacities for intrinsic motivation are associated with the number of targets within the striatum for dopamine to act upon. More recently, Gyurkovics et al. (2016) found that carriers of a genetic polymorphism that affects striatal D2-receptor availability were more prone to experience flow during study- and work-related activities. Altogether, it would thus seem reasonable to forward the initial working hypothesis that dopamine is a key substrate of intrinsic motivation.

Dopamine neurons originate in the midbrain and have two modes of activity, tonic and phasic (Grace, 1991). In the tonic mode, the neurons exhibit a steady baseline rate of firing in which dopamine is steadily released to target structures. This tonic activity promotes the normal functioning of relevant neural circuits (Schultz, 2007) and may reflect the general strength of animals’ exploratory SEEKING tendencies (Alcaro and Panksepp, 2011). In the phasic mode, dopamine neurons exhibit short bursts of activity or inactivity (above or below their baseline) in response to specific events, resulting in an increase or decrease of dopamine in target structures lasting several seconds. The phasic mode of dopamine transmission may “transiently activate SEEKING patterns in coincidence with specific cue- or context-dependent information, attributing to such information an incentive motivational, action-orienting effect” (Alcaro and Panksepp, 2011, p.1810). Of course, the tonic and phasic modes of dopamine transmission likely interact in complex ways to regulate intrinsic motivation. For example, Alcaro et al. (2007) advanced the hypothesis that moderately high levels of tonic dopamine optimize the SEEKING behavior promoted by phasic dopamine release: when tonic levels of dopamine are too low, phasic signals lack the efficacy to promote exploration; but when tonic levels are too high, phasic signals lose their informational value and exploratory behavior patterns are uncoupled from relevant contextual stimuli. Given the nascent state of the field, however, questions about how the tonic and phasic modes of dopamine release interact to influence intrinsic motivation remain outside the scope of the present effort. We instead focus on making the less specific case for a general relation between dopamine and intrinsic motivation.

Bromberg-Martin et al. (2010) recently proposed a model of dopaminergic function that is based on the recognition of two types of dopamine neurons that exhibit distinct types of phasic activity: *value-coding* neurons and *salience-coding* neurons. We review the properties of these neurons and their relevance to intrinsic motivation below.

Value-coding neurons are phasically excited by unexpected rewarding events and inhibited by unexpected aversive events; events that are wholly expected elicit little or no response. Value-coding dopamine neurons are found in the ventromedial substantia nigra pars compacta (SN) and throughout the VTA. From these midbrain regions, these neurons project axons that innervate the NAcc shell, the dorsal striatum (caudate and putamen), and the VMPFC, where they send signals about the availability of rewards, evaluation of outcomes, and learning. The phasic signals emitted by value-coding neurons are classically recognized as “reward-prediction errors” within neobehaviorist theories and are believed to be an important mechanism through which animals learn about external reinforcement contingencies (Schultz, 2007).

However, Tricomi and DePasque (2016) recently argued that, even in the absence of external rewards, this dopaminergic pathway registers the endogenous signals of positive and negative feedback that are elicited during the performance of many activities. The types of activities that people find intrinsically motivating provide just-manageable challenges, clear proximal goals, and immediate feedback (Nakamura and Csikszentmihalyi, 2014; Ryan and Deci, 2017). For example:

As people work on crossword puzzles, they get feedback from the task itself (i.e., the letters fit), and they are likely to feel a sense of joy from making progress at puzzles that challenge them…No external feedback is required, and, surely, the task-inherent positive feedback is gratifying and helps sustain interest and persistence (Ryan and Deci, 2017, p.154).

Another way of describing this optimally challenging nature of intrinsically motivated activities is to say that the positive and negative feedback that people receive during their performance of such activities is not entirely unexpected—a performative context that suggests phasic dopaminergic signaling. Following Tricomi and DePasque (2016), we therefore propose that a high rate of dopaminergic signaling within the value system is inherent to the performance of intrinsically motivating activities.

In addition to value-coding neurons, Bromberg-Martin et al. (2010) identified salience-coding neurons. These dopamine neurons are phasically excited by both unexpected rewarding and punishing events. These neurons are found in the dorsolateral SN and medial VTA, and project to the NAcc core, the dorsal striatum, and the dorsolateral PFC (DLPFC). The regions innervated by salience-coding neurons support the orienting of attention, cognitive processing, and the invigoration of actions. Dovetailing Loewenstein’s (1994) information-gap hypothesis of curiosity, DeYoung (2013) proposed that salience-related dopaminergic activity energizes exploration “in response to the incentive value of the possibility of gaining information—that is, it drives curiosity and the desire for information” (p.4). Curiosity and interest are of course long recognized components of intrinsic motivation. For example, the Intrinsic Motivation Inventory (Ryan et al., 1983), a self-report measure of intrinsic motivation for experimental settings that is used to predict free choice behavioral persistence, includes items such as “I found the task very interesting” and “I thought the task was very boring (reverse scored)”. These items describe the type of eager attentiveness and behavioral engagement that may be associated dopaminergic salience signaling. Thus, building on DeYoung (2013), we propose that the salience-coding system also subserves intrinsic motivation.

Apart from the aforementioned studies by de Manzano et al. (2013) and Gyurkovics et al. (2016), empirical studies have not directly examined the link between dopamine and intrinsic motivation. However, if intrinsic motivation is associated with dopaminergic transmission, then intrinsically motivated activities should be associated with activation across core regions of the dopaminergic systems identified by Bromberg-Martin et al. (2010). In the paragraphs that follow, we focus on neuroimaging findings relating intrinsic motivation to activity within regions of the dopaminergic value system. Studies relating intrinsic motivation to activity within regions of the dopaminergic salience system are reviewed separately because such findings are also consistent with the complementary proposition that intrinsic motivation is associated with patterns of activity across specific large-scale neural networks.

Murayama et al. (2010) examined the neural correlates of the undermining effect using fMRI. University undergraduates were asked to play a game-like stopwatch task in which they were asked to press a button within 50 ms of the 5 s mark. In a series of pilot tests, the authors determined that students found this task challenging and interesting, and therefore suitable for examining intrinsic motivation. Like classic studies on the undermining effect (e.g., Deci, 1971), participants were divided in two groups: a reward group that received performance-contingent rewards for each successful trail and a control group that received no payments. During an initial scanning session, participants in both groups evidenced greater activity in the midbrain and caudate upon the receipt of success feedback relative to failure feedback. Subsequent to the experimental manipulation, and consistent with previous behavioral studies on the undermining effect, participants in the reward group were less likely to voluntarily engage with the task during a free-choice time period relative to those in the control group. Importantly, this behavioral undermining of intrinsic motivation was paralleled by reduced activity in the caudate and midbrain during a second scanning session when monetary rewards were no longer administered to the reward group. In contrast the unrewarded group maintained its previous levels of activation. This difference in activity between the control and experimental groups is consistent with the idea that the dopaminergic value system is responsive to cues that signal task-related progress during intrinsically motivated activities.

In a more recent fMRI study, Murayama et al. (2015) had participants perform an adapted version of the stopwatch task (Murayama et al., 2010) in two conditions: an autonomy condition in which they were free to choose the appearance of the stopwatch according to their preferences and a forced-choice condition in which they had to proceed with the stopwatch selected by the computer. Results indicated that activity within the VMPFC (bilateral gyrus rectus and medial orbitofrontal gyrus) was greater upon the receipt of success feedback than failure feedback. However, this effect was modulated by the type of the trial conditions. On the one hand, the VMPFC exhibited similarly high levels of activity across success and failure feedback after free-choice (autonomy) trials. On the other hand, this region exhibited marked reductions in activity after forced-choice trials. Importantly, this sustained activity within the VMPFC in response to failure feedback was associated with enhanced performance within the free-choice condition. Present evidence suggests that value coding dopamine neurons in the midbrain project to the VMPFC and that this structure is involved in learning from negative reward prediction errors and updating outcome expectations during learning (Bromberg-Martin et al., 2010). These results are thus consistent with the idea that intrinsic motivation, and the perceived autonomy that phenomenally supports it, is associated with activity within the dopaminergic value system.

Conceptually related to these fMRI studies is research examining intrinsic motivation using electroencephalography (EEG). Two specific EEG waveforms that have been associated with intrinsic motivation are the “error-related negativity” (ERN) and the “feedback-related negativity” (FRN). Both of these waveforms are negative-going deflections in EEG recordings that arise during speeded-response tasks. Whereas the ERN appears within 100 ms following the commission of errors, the FRN appears between 200 ms and 350 ms following the receipt of negative feedback. Holroyd and Coles (2002) proposed that both the ERN and FRN arise as a consequence of phasic reductions in midbrain dopaminergic signaling to ACC, the purported neural generator of these waveforms. These phasic reductions in dopamine transmission to the ACC, and the consequent ERN and FRN, are believed to constitute a learning signal that tunes the ACC to optimize behavioral performance, an account that parallels the reward-prediction signaling of value-coding dopamine neurons (Schultz, 2007; Bromberg-Martin et al., 2010).

In a sample of school children, Fisher et al. (2009) found that intrinsic academic motivation was associated with larger ERN amplitudes during a flanker task. In a study that paralleled the design of Murayama et al. (2010), Ma et al. (2014) found that participants who had received performance-contingent monetary rewards while performing a challenging activity evidenced reduced FRN amplitudes whereas those in a control group evidenced consistently pronounced FRNs. In another study, this time paralleling the design of Meng and Ma (2015) and Murayama et al. (2015) found that having the opportunity to exercise choice during the performance of an intrinsically motivating task was associated with larger FRN amplitudes. An important caveat to these studies is their small sample sizes (*N* = 17, 36 and 18, respectively), which raises uncertainty about reliability of their reported effects. To this point, Jin et al. (2015; *N* = 16) found lower FRN amplitudes when participants received negative feedback on a supposedly interesting task relative to a boring task. In light of these small sample sizes and diversity of findings, it is clear that more decisive larger-sample studies are required. Nevertheless, the available evidence from these EEG studies is generally consistent with the idea that intrinsic motivation is associated with dopaminergic signaling.

Other evidence of a link between intrinsic motivation and the dopaminergic system comes from studies examining the neural correlates of curiosity. Kang et al. (2009; Study 1) used fMRI to examine curiosity as it is framed by the information-gap theory of Loewenstein (1994). We previously pointed out that the information-gaps in people’s knowledge structures, and the ensuing feelings of curiosity that such gaps elicit, can be productively framed in terms of people’s orienting toward optimal challenges. Participants reflected upon a series of trivia questions (e.g., What instrument was invented to sound like a human singing?) and rated their curiosity for each one. During the presentation of the trivia questions, items that elicited greater curiosity were associated with activations in the left caudate and parahippocampal gyri (PHG). Furthermore, when trivia answers were revealed following incorrect responses, participants’ level of curiosity was associated with greater activity in the midbrain and left PHG. Although Bromberg-Martin et al. (2010) did not identify the PHG as a core component of the dopamine system, Kang et al. (2009) point out that this region is involved in successful memory encoding and its activity during states of curiosity is therefore consistent with the idea that intrinsic motivation is associated with enhanced learning.

A follow-up study by Gruber et al. (2014) more directly assessed the relation between curiosity and learning. This study used trivia questions similar to Kang et al. (2009) to examine if states of curiosity improved memory for task-relevant information and for information that was incidental to the main task. Incidental information consisted of face stimuli that were presented to participants when they anticipated trivia answers. During the presentation of trivia questions, curiosity was associated with activity in the left SN/VTA, bilateral NAcc, and bilateral dorsal striatum. Furthermore, replicating the behavioral results of Kang et al. (2009; Study 2), in both immediate and delayed memory tests, participants recalled more answers for high- relative to low-curiosity questions. Extending these previous behavioral findings, Gruber et al. (2014) also found enhanced recall of incidental face stimuli presented during high-curiosity questions. These memory effects were associated with greater activity in the SN/VTA and the hippocampus during the presentation of trivia questions and increased functional connectivity between these regions when participants anticipated answers to the trivia questions.

### Proposition II: Intrinsic Motivation Entails Dynamic Switching between Brain Networks for Salience Detection, Attentional Control and Self-Referential Cognition

A complementary approach to theorizing about the neural systems that support intrinsic motivation is to map its phenomenology with the activity of large-scale neural networks (Ryan and Di Domenico, 2016). Research on structural and functional brain organization has revealed multiple large-scale brain networks that support various cognitive functions (Bressler and Menon, 2010). Among these is the so-called *salience network*, which is believed to support the detection of subjectively important events and the mobilization of attentional and working memory resources in the service of goal-directed behavior (Menon and Uddin, 2010; Menon, 2015). The salience network is anchored in the anterior insula (AI) and dorsal ACC and includes major subcortical nodes in the amygdala, NAcc, the SN, and VTA. These subcortical nodes are believed to send signals about the motivational significance of stimuli to the AI; the AI in turn is believed to integrate this information with incoming sensory inputs from both the external environment and the viscera for the bottom-up detection of contextually important events. Through its reciprocal connections with the dACC, a key structure for executive control, the AI is believed to selectively amplify neural signals of important events for the effective deployment of cognitive resources.

Little is presently known about the specific role of dopamine in the functioning of the salience network. However, AI does receive inputs from the amygdala, the likely source of the motivational salience signals sent to dopamine neurons in the midbrain, from the ventral striatum, which receives dopaminergic projections from the midbrain, and from the SN and the VTA, the midbrain regions from which dopamine neurons originate (Bromberg-Martin et al., 2010; Menon and Uddin, 2010; Menon, 2015). Additionally, the AI has reciprocal connections with the dACC, which likely receives direct input from both value- and salience-coding dopamine neurons (Bromberg-Martin et al., 2010). These connections imply that the AI may play a role in contextualizing the signals of motivational significance transmitted by both value- and salience-coding dopamine neurons. Most relevant in this regard is the suggestion that the AI functions as a dynamic hub for modulating the activity of two other large-scale brain networks (Menon and Uddin, 2010; Menon, 2015). The first, known as the *default mode network*, has major nodes in the MPFC and the posterior cingulate cortex (PCC). These regions show high levels of activity during passive resting states (Gusnard and Raichle, 2001), tasks involving internally-focused, self-referential cognition (Northoff et al., 2006), and mind-wandering (Mason et al., 2007). The second, known as the *central executive network*, includes the DLPFC and the posterior parietal cortex (PPC). The regions of this network, which are important substrates of working memory and executive functions, typically show elevated activity during cognitively demanding, externally-focused tasks. Importantly, activity across the default mode and central executive networks often fluctuates in an antagonistic manner, such that activity in one is often accompanied by suppressed activity in the other.

The antagonistic dynamic between the default mode and central executive networks, along with the role of the salience-mediating switching instigated by the AI, may inform three characteristics of intrinsic motivation. First, in its most experientially abundant state, intrinsic motivation entails cognitive absorption and non-self-conscious enjoyment of an activity (Csikszentmihalyi, 1990; Nakamura and Csikszentmihalyi, 2014). This phenomenology suggests diminished activity within regions of the default mode network, which are commonly activated during self-focused mental activity (e.g., self-reflection, rumination) and mind-wandering, and heightened activity within the central executive network, which is engaged during bouts of externally focused attention. Second, intrinsic motivation is reliably associated with enhanced performance, cognitive flexibility, and deeper conceptual learning (e.g., Grolnick and Ryan, 1987). This relation between intrinsic motivation and enhanced task performance is consistent with, and may be partly explained by, greater mobilization of the central executive network during intrinsically motivating tasks (Ryan and Di Domenico, 2016). Third, classic perspectives that describe autonomy or authenticity as a state of “organismic congruence” (e.g., Rogers, 1961) characterize it as an embodied cognitive process whereby sensory and visceral information is permitted to access and direct one’s attention, in a bottom-up manner, to events of subjective importance and meaning (also see Peterson, 1999). The salience network, and the AI most specifically, with its receipt of sensory and visceral input and its interoceptive functions (Craig, 2009; Menon and Uddin, 2010; Menon, 2015), would seem well-suited to support this aspect of autonomy, especially during intrinsic motivation when people orient themselves to stimuli that spontaneously grip their attention and interest.

Neuroimaging studies have reported patterns of neural activity consistent with the idea that intrinsic motivation recruits the salience and central executive networks, while suppressing the default mode network. In the aforementioned study by Murayama et al. (2010), the undermining of intrinsic motivation was associated with decreases in lateral PFC activity in response to task onset cues. The study by Murayama et al. (2015) found increased activity within the midbrain, ACC, and bilateral insula in response to free-choice (autonomy) cues relative to forced-choice cues at the onset of task trials. The curiosity studies by Kang et al. (2009) and Gruber et al. (2014) found greater activity within the lateral PFC during curiosity-inducing questions. More recently, Marsden et al. (2015) observed neural activations within several structures that comprise the SN. Specifically, their study found participants who spent more free-choice time solving remote-associate word problems (i.e., a behavioral marker of intrinsic motivation) showed greater activity in the ACC, amygdala, anterior and posterior insula, PHG, and caudate nucleus after trial onsets that immediately followed negative feedback for preceding trials. Jepma et al. (2012) examined the neural correlates of perceptual curiosity. Participants viewed blurry images of otherwise easily recognizable objects that induced feelings of curiosity, and were subsequently shown clear images of the objects to satisfy their curiosity. Results indicated that induction of curiosity was associated with significant activations within the AI and ACC, the core regions of the salience network, and significant deactivations within regions associated with the default mode network. Additionally, this study found that the resolution of perceptual curiosity was associated with activity within the left caudate, putamen, and NAcc, regions that comprise the core of the dopaminergic system.

A set of studies (Lee et al., 2012; Lee and Reeve, 2013) examined the neural correlates of intrinsic motivation by comparing patterns of neural activity when undergraduate students imagined themselves performing intrinsically motivating activities (e.g., “writing an enjoyable article”) and extrinsically motivating activities (e.g., “writing an extra-credit article”). Most prominently, these studies found preferential activity within insular regions when participants imagined the enactment of intrinsically motivating activities. Building on this initial work, Lee (2016) more recently described the results of an fMRI study that examined functional connectivity between striatal regions and the AI when participants attempted trivia questions and anagrams. Results indicated that when participants worked on intrinsically motivating problems (curiosity inducing-questions and competence-enabling anagrams) they evidenced greater activity and functional connectivity between these regions.

Klasen et al. (2012) examined the neural correlates of flow using fMRI recordings obtained during free play of a video game. The authors developed an objective coding system for examining different components of the flow experience based on player-generated video game contents. Consistent with the idea that intrinsic motivation is associated with dopaminergic signaling, optimal challenge was associated greater activity within the caudate, putamen, and NAcc. Consistent with the idea that intrinsic motivation is associated with suppressed activity in default mode regions, concentrated focus and goal clarity were associated with reduced activity within the orbitofrontal cortex and ACC. Additionally, task-related failure was associated with increased activity within the cuneus, a structure included within the default mode network.

In another fMRI study, Ulrich et al. (2014) examined the neural correlates of flow by asking participants to work on mental arithmetic task and comparing experimentally challenging levels with boredom and overload conditions. Results indicated that flow states were associated with increased activity in the left putamen and left IFG, again implicating core regions of both the dopaminergic system and the central executive network. Results also indicated that flow was associated with deactivations within the MPFC, suggesting suppressed default mode network activity. In another study, Yoshida et al. (2014) used functional near-infrared spectroscopy (fNIRS) to examine the time course of neural activations within the prefrontal cortex during states of flow and boredom when participants played Tetris^®^. Again, consistent with the idea that intrinsically motivated states recruit central executive regions, results indicated increasing bilateral activity within lateral PFC regions during flow. However, a subsequent fNIRS study by Harmat et al. (2015) that compared prefrontal activity across easy, optimally challenging, and difficult levels of Tetris did not any differences. Despite these mixed findings, the results of existing studies altogether suggest that future research would benefit by explicitly testing the proposition that intrinsic motivation is associated with patterns of activity across the salience, central executive, and default mode networks.

## Discussion

Recent years have witnessed an emerging interest in the neurobiological systems that support intrinsic motivational processes. Although this area of inquiry is young, conceptual and empirical evidence points to the role of dopaminergic systems in supporting intrinsically motivated behaviors. Across different mammalian species, there appear to be linkages between dopamine and the positive experiences associated with exploration, new learning and interest in one’s environment (Panksepp, 1998; Panksepp and Biven, 2012). Building on Bromberg-Martin et al.’s (2010) distinction between dopaminergic value- and salience-coding and on previous work respectively mapping these systems onto the phenomenology of competence (Tricomi and DePasque, 2016) and interest (DeYoung, 2013), we propose that intrinsic motivation entails both types dopaminergic transmission. Because these dopamine systems entail distinct neural structures, future neuroimaging studies have a strong conceptual basis for specifying distinct *a priori* regions of interest. Beyond that, evidence suggests that intrinsic motivation involves alterations between the neural networks of salience detection, attentional control, and self-referential cognition (Menon and Uddin, 2010; Menon, 2015). Better understanding of these large-scale neural dynamics may provide greater resolution of the processes that support high quality learning and performance.

Despite the clear conceptual relationship between intrinsic motivation and dopaminergic transmission, only two existing studies provide direct evidence of an association between these two processes (de Manzano et al., 2013; Gyurkovics et al., 2016). The bulk of existing research provides indirect support to the hypothesis that dopamine is a substrate of intrinsic motivation in that the core regions innervated by dopamine neurons are activated during intrinsic motivation. Pharmacological manipulations of dopamine thus represent an important new research direction. Indeed, such manipulations have already been fruitfully applied in the study of dispositional traits (e.g., Wacker and Smillie, 2015) and their application in the study of motivational states would seem a natural extension. Pharmacological manipulations of dopamine may, for example, allow researchers to more precisely decode the neural mechanisms that mediate the undermining effect of externally contingent rewards on intrinsic motivation.

The link between dopaminergic systems and intrinsic motivation may also prove useful for developmental roboticists for whom the topic of intrinsic motivation has recently fallen into purview (e.g., Gottlieb et al., 2013, 2016). The stated goal of developmental robotics is to design embodied agents that self-organize their development by constructing sensorimotor, cognitive, and social skills over the course of their interactions with the environment. Roboticists have proposed that in order for embodied agents to be capable of intrinsic motivation, they must not only be outfitted with computational systems that orient them toward novel, surprising, or uncertain stimuli, but also with meta-monitoring processes that track their learning progress in their investigation of such stimuli (Gottlieb et al., 2013, 2016). Without meta-monitoring processes that track learning, agents will likely get trapped investigating stimuli that are random or otherwise unlearnable, precluding the possibility for self-directed development. The existence of salience- and value-coding dopaminergic systems, respectively capable of tracking novelty and rewarding feedback, may partially represent an organic instantiation of the type of computational system that Gottlieb et al. (2013, 2016) hypothesize to be a requirement for intrinsic motivation. We believe that roboticists are well-positioned to discover the types of computational problems that need to be solved for a full understanding of the neural substrates of intrinsic motivation. We thus hope that some of the present ideas will help to spur robotics research on intrinsic motivation.

Future studies are also needed to directly test the hypothesis that intrinsically motivated states entail dynamic switching between the salience, central executive and default mode networks. Beyond traditional fMRI analyses comparing activity in *a priori* regions across intrinsically and non-intrinsically motivated states, this hypothesis specifically encourages the use of connectivity analyses and the adoption of chronometric techniques that can provide information about the dynamics and directionality of activity across large-scale networks (e.g., Sridharan et al., 2008). This research direction may help to not only elucidate the neural basis of intrinsic motivation but also to identify the neural mechanisms through which intrinsic motivation enhances learning and performance outcomes, especially on tasks that require depth of processing and high-quality engagement.

### Beyond Exploration, Curiosity and Mastery: Intrinsically Motivated Social Play

SDT uses intrinsic motivation as a broad term for diversity of activities that are inherently rewarding and growth promoting (Ryan and Deci, 2017). This is a large class of behaviors, minimally including curious exploration and mastery tendencies, on the one hand, and social play, on the other (Ryan and Di Domenico, 2016). To date, human neuroscience studies have focused on intrinsic motivation associated with curious exploration and mastery, rather than social play, and we accordingly based our review on this subset of intrinsically motivated behaviors. Yet, comparative affective neuroscience suggests that exploration and social play have both distinct and overlapping neurobiological and phenomenological underpinnings, the former being subserved by the SEEKING system and the latter by the *PLAY system* (Panksepp, 1998; Panksepp and Biven, 2012). The subcortical PLAY system governs the rough-and-tumble (R&T) interactions of mammals, energizing them to develop and refine their physical, emotional, and social competencies in a safe context (Panksepp, 1998; Pellis and Pellis, 2007; Trezza et al., 2010; Panksepp and Biven, 2012). In early mammalian development, R&T play constitutes a type of embodied social cognition that provides a basis for cooperation and the adaptive self-regulation of aggression (Peterson and Flanders, 2005). Humans are of course also capable of more sophisticated forms of play beyond R&T such as common playground games, sports play and friendly humor, but such human play may be nonetheless organized around basic PLAY motivations (Panksepp, 1998; Panksepp and Biven, 2012).

We might therefore regard play as intrinsically motivated socialization (Ryan and Di Domenico, 2016), an expression of people’s complementary tendencies toward autonomy and sociality in development (Ryan, 1995; Ryan et al., 1997). Indeed, research in SDT suggests that in addition to competence and autonomy, people have a basic psychological need for *relatedness*, the sense of feeling meaningfully connected with others (Ryan and Deci, 2017). Whereas strong associations between exploratory intrinsic motivations and satisfactions of competence and autonomy have been clearly demonstrated, relatedness is usually seen to play a more distal role in the expression of these intrinsic motivations. Specifically, relatedness satisfactions provide people (especially children) with a sense of safety, a secure base from which their exploratory tendencies can be more robustly expressed (Ryan and Deci, 2017). Recognition of social PLAY signifies the centrality of the need for relatedness in some intrinsically motivated activities.

Interest in the overlaps and contrasts between intrinsically motivated exploration and play is thus an important agenda for future studies and both are relevant to intrinsic motivation as it is studied within SDT (Ryan and Di Domenico, 2016). Behavioral models of human intrinsic motivation have generally conflated exploration and play because these activities share common features such as an internal perceived locus of causality and perceived competence or mastery. Indeed, functional distinctions between intrinsically motivated exploration and object or manipulative play are subtle and suggest that, for many activities recognized as “playful”, the conflation is appropriate and productive. For example, Wilson (2000) suggested that “In passing from exploration to play, the animal or child changes its emphasis from ‘What does this object do?’ to ‘What can *I* do with this object?”’ (p.165). In fact, intrinsically motivated object play, manipulative play, and solitary gaming likely arise from the activity of the SEEKING system (Panksepp, 1998; Panksepp and Biven, 2012). Clearly, more empirical work is needed to differentiate these types of intrinsic motivation in humans.

### Methodological Suggestions

Our principal intent in this review article, is to stimulate increasing integration between social behavioral research on intrinsic motivation and the neuroscience of motivation. We see many new and promising pathways opening up. At the same time, methodological issues persist that warrant serious considerations. We list but a few of these.

First, intrinsic motivation and the associated undermining effect of rewards on these behaviors pertain only to tasks that are interesting and enjoyable in the first place. Thus, researchers should pilot test target activities to ensure that the activities are suitable for examining the undermining effect. This is especially important in neuroscience, where contemporary methods such as fMRI often involve procedures that limit how interesting experimental tasks can be. Researchers should also use multi-method assessments of intrinsic motivation to validate their measures and to ensure that the correct behavioral phenomena are being tapped. For example, in an attempted (and failed) replication and extension of Murayama et al.’s (2010) fMRI study on the undermining effect, Albrecht et al.’s (2014) utilized a picture-discrimination task for which participants may not have been intrinsically motivated in the first place (pilot testing was not reported) and for which free-choice behavior was not examined as a dependent variable. In the absence of these important design characteristics, it is difficult to draw decisive conclusions from their experiment. Incidentally, we note that Albrecht et al.’s (2014) study did show that competence feedback increased participants’ self-reported fun and that it was also associated with increased activations within the midbrain, striatum, and lateral PFC, findings that are consistent with the idea that competence is associated with dopamine-related activity.

Second, replicability is a central concern, as it is throughout the social and personality neurosciences (Allen and DeYoung, 2016). Most studies to date have been small-sample investigations, and larger samples are needed if we are to derive foundational conclusions. *A priori* hypotheses concerning regions of interest will also add confidence to the interpretation of findings. Toward that end, the present review ought to provide future studies with a useful reference for making clear predictions about the neural basis of intrinsic motivation.

### Conclusion

Intrinsic motivation is a topic of interest within both basic behavioral science and applied translational studies and interventions (Ryan and Deci, 2000, 2017). Yet important to the progress of empirical research on intrinsic motivation is integrating what is known from phenomenological and behavioral studies with neuroscience studies. As we suggested at the outset, neuroscience holds potential for testing existing models of the situational and social determinants of intrinsic motivation as well as for providing greater resolution on the affective and cognitive processes that underpin such activities. Movement toward consilience is a central concern to SDT and our hope is that the current synthesis provides some broad stoke encouragement for that agenda.

## Author Contributions

SID conceptualized and wrote the manuscript. RMR assisted in conceptualizing and writing the manuscript.

## Funding

SID was supported in this research by a postdoctoral fellowship from the Social Sciences and Humanities Research Council of Canada.

## Conflict of Interest Statement

The authors declare that the research was conducted in the absence of any commercial or financial relationships that could be construed as a potential conflict of interest.
